# Emerging trends in antipsychotic and antidepressant drug development: Targeting nonmonoamine receptors and innovative mechanisms

**DOI:** 10.1002/pcn5.157

**Published:** 2023-11-20

**Authors:** Hitoshi Osaka, Tetsufumi Kanazawa

**Affiliations:** ^1^ Department of Neuropsychiatry Osaka Medical and Pharmaceutical University Takatsuki‐city Osaka Japan

**Keywords:** antidepressant, antipsychotic, Auvelity, nonmonoamine, ulotaront

## Abstract

The domain of psychiatric drug development is currently witnessing a notable transformation, with a paramount emphasis on targeting nonmonoamine receptors and exploring inventive mechanisms of action. This paper presents an overview of the ongoing advancements in antipsychotic and antidepressant drug development. Historically, antipsychotics predominantly targeted dopamine receptors, but there is now an escalating interest in drugs that act on alternative receptors, exemplified by the TAAR1 receptor. One noteworthy candidate is Ulotaront (SEP‐363856), an agent acting as a TAAR1 agonist with 5‐HT1A agonist activity, demonstrating promising outcomes in the treatment of schizophrenia, devoid of extrapyramidal symptoms or metabolic side‐effects. Similarly, MIN‐101 (Roluperidone) and KarXT are currently in development, with its focus on addressing the symptoms in schizophrenia. In the domain of antidepressants, novel therapeutic approaches have surfaced, such as Auvelity, a Food and Drug Administration (FDA)‐approved NMDA receptor antagonist synergistically combined with Bupropion to enhance its effects. Another notable candidate is Zuranolone, operating as a GABA A receptor‐positive allosteric modulator, showcasing efficacy in treating major depressive disorder (MDD) and postpartum depression. Additionally, TAK‐653 (NBI‐1065845) and MJI821 (Onfasprodil) have emerged as potential antidepressants targeting AMPA receptors and NMDA receptor 2B (NR2B) negative allosteric modulation, respectively. This paper underscores the transformative potential of these novel drug candidates in psychiatric treatment and their ability to address cases that were previously treatment‐resistant. By focusing on nonmonoamine receptors and introducing innovative mechanisms, these drugs offer a promising prospect of improved outcomes for individuals suffering from schizophrenia and MDD. Thus, sustained attention and dedication to the development of such drugs are essential to augmenting the therapeutic options available for psychiatric patients.

## INTRODUCTION

While various treatments exist for psychiatric disorders, such as psychotherapy and neuromodulation therapy, drug treatment remains the predominant approach.^1^, ^2^ Over the last approximately 50 years, these drugs have undergone significant development within their respective classes. Notably, antipsychotic medications were primarily used for schizophrenia but have recently found expanded clinical applications in bipolar disorder, autism, and agitation related to dementia. Similarly, antidepressants were primarily utilized for depression, but their indications have been broadened to include various anxiety disorders. Mechanistically, antipsychotics primarily act through dopamine blockade, supplemented with monoamines like serotonin, thus contributing to the reduction of adverse effects. Likewise, antidepressants exert their effects by modulating serotonin, noradrenaline, and other monoamines in the synaptic cleft.

Beyond these mechanisms, we are currently witnessing an exciting era of testing new drugs for marketing. In the following sections, we provide an overview of the two drug classes mentioned above and highlight some of the drugs anticipated to be launched in the near future. We have compiled a list‐as exhaustive as possible‐of drugs that have advanced to Phase 3 in major countries, with additional noteworthy drugs also in Phase 2.

These novel mechanisms of action hold the potential to revolutionize the treatment response for psychiatric disorders and offer therapeutic options for patients who have previously shown inadequate responses to conventional treatments.

## SURVEY METHODOLOGY

In this survey, our primary focus was on two drug categories – antipsychotics and antidepressants – aiming to assess the current state of drug development. The survey was conducted primarily by exploring the websites of pharmaceutical manufacturers, utilizing keywords such as “new drug development status,” “pipeline,” and “financial reports.” We deliberately opted not to rely on articles from PubMed, which are commonly used for systematic reviews, as they might entail information delays due to the peer‐review process. Consequently, the data presented in this review have not undergone complete scientific verification and should be regarded solely as a point of reference.

We provide an overview of antipsychotic drugs currently in development (Table 1), encompassing a summary of drugs that have advanced to Phase 3 in major countries and Phase 2 drugs warranting attention. Furthermore, the review discusses specific antipsychotic drugs, such as SEP‐363856 (Ulotaront) and MIN‐101 (Roluperidone), that are likely to garner considerable attention in the future. Additionally, we include a similar analysis for antidepressants currently under development (Table 2), featuring drugs such as Auvelity and Zuranolone, which are poised to be significant contributors in the field.

**Table 1:** Antipsychotic drugs in development.

Drugs in development	Mechanisms	Expected effects	Current status of research (from https://clinicaltrials.gov/)	Current status of research (from PMDA)
SEP‐363856 Ulotaront	TAAR1 and 5‐HT1A agonist	Improvement of positive and negative symptoms of schizophrenia	US Phase 2: 2 completed US Phase 3: 1 completed, 3 in progress Japan Phase 3: 1 in progress	Japan Phase 3: in progress
MIN‐101 Roluperidone	Serotonin/sigma receptor/α‐adrenergic receptor antagonist	Improvement of negative symptoms of schizophrenia	US Phase 3: 1 completed	N/A
KarXT xanomeline‐trospium	M 1/M 4 preferred muscarinic agonist	Schizophrenia	US Phase 2: 1 completed US Phase 3: 3 completed, 5 in progress	N/A
MK‐8189	Phosphodiesterase 10 A inhibitor (PDE10 inhibitor)	Schizophrenia	US Phase 3: 1 completed, 1 in progress	N/A
RG7906 RALMITARONT	TAAR1 partial agonist action	Schizophrenia	US Phase 2: 2 completed	N/A
F17464	D3 antagonist/5‐HT partial agonist	Schizophrenia	US Phase 2: 1 completed	N/A

Abbreviation: PMDA, Pharmaceuticals and Medical Devices Agency.

**Table 2:** Antidepressant drugs in development.

Drugs in development	Mechanisms	Expected effects	Current status of research (from https://clinicaltrials.gov/)	Current status of research (from PMDA)
Auvelity	NMDA receptor antagonist and Bupropion in an extended‐release combination	Antidepressant effect in 1 week	US Phase 2: 4 completed US Phase 3: 3 completed	N/A
Zuranolone	GABA A receptor positive allostatic modulator	Antidepressant effect	US Phase 2: 1 completed US Phase 3: 7 completed	Japan Phase 3: in progress
TAK‐653	AMPA receptor selective positive allosteric modulator	Major depression, treatment‐resistant depression	US Phase 2: 1 in progress	N/A
MIJ８２１	NMDA receptor 2B negative allostatic modulator	Major depression, treatment‐resistant depression	US Phase 2: 1 completed, 1 in progress	N/A
Seltorexant	Orexin‐2 receptor antagonist	Improvement of major depressive and insomnia symptoms	US Phase 2: 1 completed US Phase 3: 2 completed, 2 in progress	N/A

Abbreviation: PMDA, Pharmaceuticals and Medical Devices Agency.

## MAJOR NEW ANTIPSYCHOTIC DRUG CANDIDATES

### SEP‐363856 (Ulotaront)

Ulotaront is a promising antipsychotic candidate acting as a trace amine‐associated receptor 1 (TAAR1) agonist with serotonin 5‐HT1A agonist activity, while lacking dopamine D2 or serotonin 5‐HT2A receptor binding. Sunovion, in collaboration with PsychoGenics, discovered this agent utilizing the in vivo phenotypic SmartCube platform and artificial intelligence algorithms. Phase 2 results in patients experiencing acute exacerbations of schizophrenia demonstrated efficacy in addressing both positive and negative symptoms of schizophrenia, with side‐effects similar to those of placebo. Particularly noteworthy is the drug's absence of extrapyramidal symptoms (EPS), weight gain, lipid or glucose changes, or prolactin elevation. In recognition of its potential, the US Food and Drug Administration (FDA) granted Ulotaront the Breakthrough Therapy designation for schizophrenia treatment in May 2019.

The Phase 2 study results of SEP‐363856 have been published in the *New England Journal of Medicine*.^3^ The study showed that treatment with SEP‐363856 (50–75 mg once daily) resulted in a statistically significant (*p* < 0.05) change in the primary endpoint of the Positive and Negative Syndrome Scale (PANSS) total score after 4 weeks of treatment, compared to placebo (*p* < 0.05). The total change in PANSS score was statistically significant (−17.2 in the Clozapine group and −9.7 in the placebo group, *p* = 0.001) and clinically meaningful, indicating that the primary endpoint was met. Furthermore, the Clinical Global Impression Scale—Severity of Illness (CGI‐S) showed improvement (*p* < 0.001) in the treatment group compared to the placebo group, and improvements (*p* < 0.02) were also observed in all PANSS subscales (Positive, Negative, and Overall Psychopathology). Throughout the study, Ulotaront exhibited a safety profile comparable to placebo in terms of EPS, body weight, and other metabolic measures, with the drug generally being well‐tolerated. In an open‐label study where Ulotaront was continuously administered for 6 months, efficacy was sustained, and no new safety or tolerability concerns were noted.^3^

TAAR1, the primary receptor targeted by the drug, is the sole endogenous receptor for trace amines like beta‐phenylethylamine (phenylethylamine [PEA]) and tyramine, which exhibit lower blood levels in humans than dopamine and noradrenaline.^4^ TAAR1 is capable of reducing the firing rate of dopamine neurons and preventing a state of dopaminergic hyperactivity through direct activation of G protein‐coupled inwardly rectifying potassium channels (GIRKs).^5^, ^6^ The global anticipation of antipsychotic drugs that do not directly target dopamine has been steadily growing.

The drug is under codevelopment with Otsuka Pharmaceutical and Sumitomo Pharma, progressing to Phase 3 in the United States and Phase 2 in Japan. Very recently, Phase 3 trials did not demonstrate statistically significant improvement compared to placebo in PANSS.^7^

### MIN‐101 (Roluperidone)

Negative symptoms represent a significant contributing factor to the impaired functional outcome observed in patients with schizophrenia.^8^, ^9^ A vast majority of individuals meeting diagnostic criteria for schizophrenia experience negative symptoms,^10^ which tend to persist throughout their lives.^11^ The severity of negative symptoms significantly influences the extent of social and occupational dysfunction in these patients.^12^

On May 1, 2023, Minerva Neurosciences Inc. provided an update on the New Drug Application (NDA) for Roluperidone (iloperidone) for treating negative symptoms in schizophrenia. The drug, currently in Phase 3 in the United States, acts by blocking serotonin, δ, and α‐adrenergic receptors involved in mood regulation, cognition, sleep, and anxiety. Its specific therapeutic focus lies in addressing negative symptoms in schizophrenic patients. It is crucial to note that there are presently no approved treatments for negative symptoms of schizophrenia in the United States.

In a study assessing the efficacy and safety of iloperidone in treating negative symptoms of schizophrenia, 513 schizophrenic patients with moderate to severe negative symptoms received iloperidone at doses of 32 mg/day, 64 mg/day, or placebo for a duration of 12 weeks. The Negative Symptom Factor Score (NSFS) for iloperidone 64 mg demonstrated a statistically significant decrease compared to placebo (*p* ≤ 0.044), albeit marginally, affirming the drug's effectiveness in alleviating negative symptoms.^13^

### KarXT

KarXT, comprising xanomeline, a muscarinic agonist with a preference for M1/M4 receptors, and trospium, a nonselective muscarinic antagonist, represents a central‐nervous‐system‐specific formulation. It aims to harness the therapeutic potential of xanomeline while mitigating the adverse effects observed in initial investigations.^14^ The activity of M1 and M4 receptors is linked to symptoms of severe neuropsychiatric conditions, such as Alzheimer's disease psychosis, positive and negative symptoms of schizophrenia, and cognitive deficits, with indirect modulation of dopamine neurotransmission in relevant brain regions.^15^, ^16^, ^17^, ^18^, ^19^, ^20^ Trospium, due to its inability to penetrate the central nervous system, serves to decrease the peripheral cholinergic side‐effects associated with xanomeline.

Currently, KARUNA is spearheading the development of KarXT, evaluating its efficacy in Phase 3 clinical trials, both as a standalone therapy and as an adjunctive treatment for schizophrenia and psychosis in Alzheimer's disease.

In a 5‐week randomized, double‐blind, placebo‐controlled Phase 2 trial involving inpatients with schizophrenia, 179 patients received at least one dose of either KarXT (*n* = 89) or placebo (*n* = 90). KarXT successfully achieved its primary efficacy endpoint and numerous secondary endpoints. Most procholinergic and anticholinergic adverse events associated with KarXT were mild and typically occurred during the initial 1–2 weeks of treatment. These symptoms were transient, with a median duration ranging from 1 day (vomiting) to 13 days (dry mouth). Notably, no patients in either treatment group discontinued the study due to procholinergic or anticholinergic adverse events. The incidence of somnolence/sedation adverse events with KarXT was low and comparable to that observed in the placebo group.^20^

According to recently published results from the Phase 3 randomized, double‐blind, placebo‐controlled EMERGENT‐2 trial, KarXT demonstrated a statistically significant and clinically meaningful reduction of 9.6 points (effect size = 0.61) in the PANSS total score from baseline to Week 5, in comparison to placebo. This significant improvement in PANSS total score was evident as early as Week 2 (the first postbaseline assessment) and persisted throughout the study. Rates of serious treatment‐emergent adverse events were comparable between the KarXT and placebo groups, with none attributed to the drug. The most prevalent treatment‐emergent adverse events (occurring in ≥5% of patients) were of mild to moderate severity and included constipation, indigestion, nausea, vomiting, headache, increased blood pressure, dizziness, gastroesophageal reflux disease, abdominal discomfort, and diarrhea.^21^

## MAJOR NEW ANTIDEPRESSANT CANDIDATES

### Auvelity

Auvelity stands as the sole FDA‐approved NMDA receptor antagonist for antidepressant use, presented as a sustained‐release fixed‐dose combination of Dextromethorphan hydrobromide and the antidepressant Bupropion. The ingenious design of Auvelity involves utilizing Bupropion, a CYP2D6 inhibitor, to elevate and prolong plasma concentrations of Dextromethorphan hydrobromide, a conventional NMDA receptor (ion channel glutamate receptor) antagonist and sigma 1 receptor agonist. Bupropion, beyond its CYP2D6 inhibition, also exhibits relatively weak inhibition of norepinephrine and dopamine neuronal reuptake, leading to additional anticipated antidepressant effects. Auvelity's mechanism of action involves the amelioration of GABA‐mediated neuronal dysfunction and modulation of glutamatergic neurotransmission through direct interaction with postsynaptic NMDA receptors.^22^, ^23^ This drug, developed by Axsome Therapeutics Inc., obtained FDA approval in August 2022 for the treatment of major depressive disorder (MDD).

In the GEMINI study, a Phase 3, double‐blind, placebo‐controlled trial, 327 patients with MDD were equally randomized to receive Auvelity or placebo, and their progress was assessed over 6 weeks. The study successfully achieved its primary endpoint, showcasing statistically significant improvement in the Montgomery–Åsberg Depression Rating Scale (MADRS) total score at Week 6. Auvelity demonstrated significant improvement in MADRS scores starting as early as Week 1 and maintaining this progress until Week 6. Additionally, Clinical Global Impression (CGI) scores reflected substantial symptom improvement at Week 1, sustaining this improvement through Week 6. Notably, CGI appears to exert a more immediate effect compared to prior antidepressants. Moreover, the percentage of patients achieving remission (MADRS total score of 10 or less) increased to 40% by Week 6.^24^

### Zuranolone

Zuranolone, a biweekly, once‐daily oral treatment, is designed for individuals with MDD. As an oral neuroactive steroid (NAS), it acts as a GABA A receptor‐positive allosteric modulator (PAM). GABA A receptors are crucial inhibitory signaling pathways in the brain and central nervous system, significantly influencing brain function regulation.^25^

Sage Therapeutics Inc., Biogen Inc., and Shionogi are collaboratively developing this drug, currently in Phase 3 trials in both the United States and Japan. Zuranolone has also garnered Breakthrough Therapy designation from the US FDA.^26^

The WATERFALL Study, a Phase 3, double‐blind, placebo‐controlled trial, assessed the efficacy and safety of zuranolone in adults with MDD. Involving 543 patients, the study assigned participants randomly to receive either 50 mg of zuranolone or placebo once nightly for 2 weeks. The primary study endpoint was the change in the 17‐item Hamilton Rating Scale for Depression (HAM‐D) total score from baseline at Day 15. The results demonstrated that the administration of zuranolone 50 mg resulted in statistically significant and clinically meaningful reductions in depressive symptoms compared to placebo (*p* = 0.0141). Furthermore, the safety of the drug has been confirmed.^27^

Zuranolone has also undergone a Phase III randomized controlled trial for investigating its efficacy and safety in treating postpartum depression. The study showed remarkable improvement starting from Day 3 of treatment, with reductions observed in HAMD‐17 and MADRS scores, which were sustained until the end of the study (Day 45). Notably, Zuranolone exhibited significant improvement compared to placebo at Day 45.^28^ For postpartum depression, brexanolone, under the trade name Zulresso, has already obtained FDA approval in 2019. It is another drug developed by Sage Therapeutics Inc., the same company behind Zuranolone, and acts as a PAM of GABA receptors. The dosing regimen involves continuous intravenous infusion over a 2.5‐day period.^29^

### TAK‐653 (NBI‐1065845)

TAK‐653 is a promising oral tablet being developed for the treatment of MDD and treatment‐resistant depression (TRD). As a selective PAM of the AMPA receptor, TAK‐653 acts to potentiate the action of agonist binding at critical sites of the AMPA receptor by slowing receptor desensitization and internal migration.^30^, ^31^, ^32^

In collaboration with Neurocrine Biosciences Inc. and Takeda, TAK‐653 is currently undergoing Phase 2 development in both the United States and Japan.^33^ This novel compound holds the potential to address MDD and TRD with its unique mechanism of action as a PAM of the AMPA receptor.

### MIJ 821 (Onfasprodil)

MIJ 821, also known as Onfasprodil, is currently under development for the treatment of TRD and MDD. This drug is administered through intravenous and subcutaneous infusions. Its mechanism of action involves functioning as an NMDA receptor 2B (NR2B) negative allosteric modulator (NAM). Allosteric modulators act at a site distinct from the orthosteric site of action of the endogenous ligand and exhibit selective binding to receptor subtypes. While positive allosteric modulators enhance the strength of the receptor's signal to the cell, NAMs like MJI821 weaken the signal.^34^

Currently being developed by Novartis Inc., MIJ 821 is in Phase 2 of clinical trials.^35^ The results from a Phase II double‐blind randomized controlled trial involving 70 patients with TRD showed that MIJ 821 at doses of 0.16 and 0.32 mg/kg, administered intravenously on a weekly or biweekly basis for 6 weeks, produced significant reductions in the MADRS total score at 24 h compared to placebo. Specifically, the MIJ 821 0.16 mg/kg pooled group exhibited a reduction of −15.51 (*p* = 0.0013), and the MIJ 821 0.32 mg/kg pooled group demonstrated a reduction of −12.98 (*p* = 0.0196).^36^ These promising results suggest the potential of MIJ 821 as a treatment option for individuals with TRD and MDD.

## CURRENT STATUS OF NEW DRUG DEVELOPMENT

The development of new antipsychotic agents for schizophrenia has primarily focused on drugs targeting dopamine D2 receptors, following the dopamine hypothesis.^37^ Additionally, efforts have been made to enhance cognitive function, increase anxiolytic and antidepressant effects, and minimize EPS and metabolic side‐effects by broadening the range of serotonin receptor affinities. Notably, Aripiprazole, Brexpiprazole, and Lurasidone have received high evaluations, particularly in terms of safety.^38^, ^39^ Recently developed antipsychotic drugs have shifted their action towards TAAR1 receptors, sigma receptors, and α‐adrenergic receptors, with a focus on receptors other than dopamine receptors. These novel agents are anticipated to yield different responses from the conventional pharmacological paradigm, potentially proving effective in patients previously considered resistant to conventional drug therapies.

Similarly, the development of novel antidepressant drugs remains a critical endeavor in managing MDD. Historically, antidepressants have been based on the monoamine hypothesis,^40^ primarily targeting serotonin and noradrenaline. However, their slow onset of action poses challenges, particularly for patients at high risk of suicide and impulsive behavior.^41^, ^42^ In recent times, agents acting on NMDA, sigma, and GABA receptors have emerged as an alternative mechanism of action compared to conventional antidepressants. This difference in mechanism holds the potential to achieve clinical efficacy earlier in the treatment process. These advancements represent significant progress in the ongoing quest for more effective and efficient treatments for schizophrenia and MDD.

While these differently acting antipsychotics and antidepressants have the potential to bring new patient populations into therapeutic range, they may also cause side‐effects due to their different framework. Therefore, Table 3 lists the major side‐effects that occurred in Phase 2 and Phase 3 of each drug. Note that TAK‐653 (NBI‐1065845) was omitted due to lack of data.

**Table 3:** Major adverse events from selected new antipsychotic/antidepressant drugs.

	Antipsychotics			Antidepressant		
Drug	Ulotaront SEP‐363856	Roluperidone MIN‐101	KarXT	Auvelity	Zuranolone	MIJ821
DOSE	50 mg or 75 mg	32 mg or 64 mg	50 mg/20 mg or 125 mg/30 mg	45 mg/105 mg	30 mg or 20 mg	0.32 mg or 0.16 mg
AE①	Somnolence 6.7%	Insomnia 10%	Constipation 21.4%	Dizziness 16%	Fatigue 6.8% 1.6%	Memory loss 42% 10%
②	Agitation 5%	Worsening of schizophrenia 8%	Dyspepsia 19%	Nausea 13%	Somnolence 6.8% 5.9%	Dizziness 11% 24%
③	Nausea 5%	Headache 5%	Nausea 19%	Headache 8%	Headache 6.3 11.2%	Feeling sleepy 21% 14%
④	N/A	N/A	Vomiting 14.3%	Diarrhea 6.8%	Dizziness 5.7% 7.4%	Headache 16% 5%
⑤	N/A	N/A	Headache 13.5%	Somnolence 6.8%	Sedation 4.7% 5.9%	Not feeling like themself 11% 19%

*Note*: Ulotaront SEP‐363856 adapted from Koblan et al.^3^

Roluperidone MIN‐101 adapted from Davidson et al.^13^

KarXT adapted from Correll et al.^21^

Auvelity adapted from Iosifescu et al.^24^

Zuranolone adapted from Clayton et al.^25^

MIJ821 adapted from the effects and safety of MIJ821 for people with treatment‐resistant depression Trial No. CMIJ821X2201 by Novartis Clinical Results Summary.^43^

Abbreviation: AE, adverse events.

## CONCLUSION

The development of new antipsychotic drugs is poised to shift its focus from drugs primarily acting on dopamine receptors to those targeting other receptors. The exploration of newly studied receptors, such as TAAR1 and others, holds the potential to enhance the treatment of negative symptoms and cognitive function in schizophrenia.

Similarly, the development of new antidepressant drugs has also been emphasizing novel mechanisms of action, including NMDA receptors, different from conventional approaches. These advancements are expected to offer promising treatment options for depression in the future. It is worth noting that a significant proportion of patients are treatment‐resistant, and a substantial number experience relapse within a year even after responding to initial antidepressant treatment. As a result, the quest for new agents that are both fast‐acting and effective for TRD remains highly desired.

Looking ahead, continued attention and focus on the development of new antipsychotic and antidepressant drugs are warranted. The potential breakthroughs in these areas can lead to improved treatment options and better outcomes for individuals living with schizophrenia and MDD.

## AUTHOR CONTRIBUTION

Hitoshi Osaka and Tetsufumi Kanazawa made substantial contributions to the study concept, drafted the manuscript, approved the final version of the manuscript to be published, and agreed to be accountable for all aspects of the work.

## CONFLICT OF INTEREST STATEMENT

Tetsufumi Kanazawa is an Editorial Board member of *Psychiatry and Clinical Neurosciences Reports* and a co‐author of this article. To minimize bias, they were excluded from all editorial decision‐making related to the acceptance of this article for publication. The authors declare no conflict of interest.

## ETHICS APPROVAL STATEMENT

N/A.

## PATIENT CONSENT STATEMENT

N/A.

## CLINICAL TRIAL REGISTRATION

N/A.

## Data Availability

N/A.
